# N-Stearidonoylethanolamine Restores CA1 Synaptic Integrity and Reduces Astrocytic Reactivity After Mild Traumatic Brain Injury

**DOI:** 10.3390/ijms27010471

**Published:** 2026-01-02

**Authors:** Anastasia Egoraeva, Igor Manzhulo, Darya Ivashkevich, Anna Tyrtyshnaia

**Affiliations:** A.V. Zhirmunsky National Scientific Center of Marine Biology, Far Eastern Branch, Russian Academy of Sciences, 690041 Vladivostok, Russia; egoraeva_a@bk.ru (A.E.); i-manzhulo@bk.ru (I.M.); owncean@yandex.ru (D.I.)

**Keywords:** mild traumatic brain injury, N-stearidonoylethanolamine, astrocytic reactivity, neuroinflammation, dendritic spine plasticity, glia-synapse interactions, anxiety-like behavior

## Abstract

Mild traumatic brain injury (mTBI) disrupts hippocampal network function through coordinated alterations in glial reactivity, synaptic integrity, and adult neurogenesis. Effective therapeutic approaches targeting these interconnected processes remain limited. Lipid-derived molecules capable of modulating these mTBI-induced disturbances are emerging as promising neuroprotective candidates. Here, we investigated the effects of N-stearidonylethanolamine (SDEA), an ω-3 ethanolamide, in a mouse model of mTBI. SDEA treatment attenuated astrocytic reactivity, restored Arc expression, and improved dendritic spine density and morphology in the CA1 hippocampal area. In the dentate gyrus, mTBI reduced Ki-67-indexed proliferation while leaving DCX-positive immature neurons unchanged, and SDEA partially rescued proliferative activity. These effects were accompanied by improvements in anxiety-like behavior and working-memory performance. Together, these findings demonstrate that SDEA modulates several key components of the glia-synapse-neurogenesis axis and supports functional recovery of hippocampal circuits following mTBI. These results suggest that ω-3 ethanolamides may represent promising candidates for multi-target therapeutic strategies in mTBI.

## 1. Introduction

Mild traumatic brain injury (mTBI) is a highly prevalent form of brain trauma and a major contributor to long-term neuropsychiatric complications, including anxiety, cognitive decline, and emotional dysregulation [1,2]. To model these processes, we employed a well-established closed-head mTBI paradigm that produces persistent functional and molecular alterations in the hippocampus without inducing gross tissue damage. This model is widely used to mimic key features of human mTBI, including long-term cognitive and emotional disturbances, glial reactivity, and synaptic dysfunction, while preserving overall brain architecture [3,4]. Despite its high incidence, the cellular and molecular mechanisms underlying persistent dysfunction after mTBI remain only partially understood, and no effective disease-modifying therapies are currently available. A growing body of evidence indicates that even mild injury triggers prolonged disturbances in hippocampal circuits, leading to alterations in glutamate homeostasis, synaptic plasticity, and neuroinflammatory signaling [3,4]. These changes are frequently dissociated from overt neuronal loss, suggesting that subtle structural and glial-mediated processes play a key role in the delayed emergence of behavioral deficits.

Astrocytes represent a central hub in this cascade. Following mTBI, astrocytes undergo pronounced reactive remodeling characterized by GFAP upregulation, NF-κB activation, cytokine release, and disruption of metabolic and glutamate-buffering functions [5,6,7,8]. This reactive state contributes to synaptic vulnerability by amplifying inflammatory signaling and impairing the stability of excitatory synapses, particularly in CA1, which is known to be highly sensitive to metabolic and inflammatory stress [9,10]. Activation of astrocytes and engagement of inflammasome pathways (multiprotein innate immune complexes that regulate maturation of pro-inflammatory cytokines) are critical mechanisms in the pathophysiology of TBI, influencing the development of secondary injury and determining long-term neurological outcomes [11]. Astrocyte-derived IL-6 can further modify PSD-95 palmitoylation (a post-translational lipid modification that regulates membrane anchoring and synaptic retention of PSD-95) and enhance the synaptic recruitment of GluR1-containing AMPA receptors, demonstrating a direct glia-to-synapse control of AMPAR localization [12]. In neuronal cultures, pro-inflammatory cytokines such as IL-1β decrease dendritic spine density and alter PSD-95 distribution without inducing neuronal death, indicating a primarily synaptic level of injury, and inflammatory molecules released from mechanically injured astrocytes similarly induce presynaptic loss in cortical networks [9,13]. Together, these studies support the view that astrocyte-driven inflammation can disrupt synaptic scaffolds, AMPA receptor organization, and activity-dependent plasticity programs such as Arc (activity-regulated cytoskeleton-associated protein, an immediate early gene critically involved in AMPA receptor trafficking and activity-dependent synaptic plasticity), thereby weakening hippocampal network connectivity even when gross neuronal survival is preserved. Parallel alterations in hippocampal neurogenesis, including reduced proliferation of dentate gyrus progenitors, further constrain recovery of circuit function after injury [14].

Against this background, lipid-derived signaling molecules capable of modulating glial reactivity and synaptic stability have attracted increasing attention as potential therapeutic candidates after mild traumatic brain injury. In particular, ethanolamides derived from ω-3 polyunsaturated fatty acids represent a biologically active class of endogenous lipid mediators with demonstrated neuroprotective and anti-inflammatory properties. Among them, ω-3 ethanolamides such as N-docosahexaenoylethanolamine (synaptamide, DHEA) and eicosapentaenoylethanolamide (EPEA) have attracted particular interest due to their anti-inflammatory, neurotrophic, and synapse-stabilizing properties [15,16,17,18,19]. Several studies also indicate that ω-3 PUFAs and their ethanolamide derivatives, such as DHEA and EPEA, can modulate neurogenic processes by improving the inflammatory and trophic milieu within the dentate gyrus [16,20], although their role in mTBI has not yet been systematically examined. To date, it remains unclear whether ω-3 ethanolamides can simultaneously modulate astrocytic reactivity, synaptic integrity, neurogenic responses, and behavioral outcomes after mTBI.

In this study we examine how N-stearidonoylethanolamine (SDEA, an ethanolamide of stearidonic acid, 18:4, n-3) influences hippocampal function after mTBI by assessing multiple levels of circuit regulation. SDEA is a less studied member of the ω-3 ethanolamide family. Although direct data on SDEA biological activity are limited, its structural similarity to other ω-3 ethanolamides suggests that it may share key functional properties, including modulation of neuroinflammatory signaling, regulation of glial reactivity, and stabilization of synaptic architecture [18,19]. Stearidonic acid serves as a metabolic precursor of longer-chain ω-3 fatty acids, and its ethanolamide derivative may act as a bioactive lipid mediator influencing glia-neuron interactions [21]. Based on the established actions of DHEA and EPEA, we hypothesized that SDEA could attenuate astrocytic activation, preserve synaptic integrity, and support activity-dependent plasticity following mTBI.

We evaluated the effects of SDEA on glial reactivity, immediate early gene activity, synaptic organization within the CA1 region and dentate gyrus, proliferative activity within the subgranular zone (SGZ) of the dentate gyrus (DG), a region associated with adult hippocampal neurogenesis, as well as behavioral performance in recognition memory, working memory, and anxiety-related paradigms. This integrative analysis combines molecular, structural, and behavioral indicators to characterize the glia-synapse-neurogenesis axis under injury conditions and identifies SDEA as a potential multi-target modulator of hippocampal recovery.

## 2. Results

### 2.1. Behavioral Outcomes: Cognitive and Emotional Consequences of mTBI and Treatment

#### 2.1.1. Elevated Plus Maze

**Figure 1:**
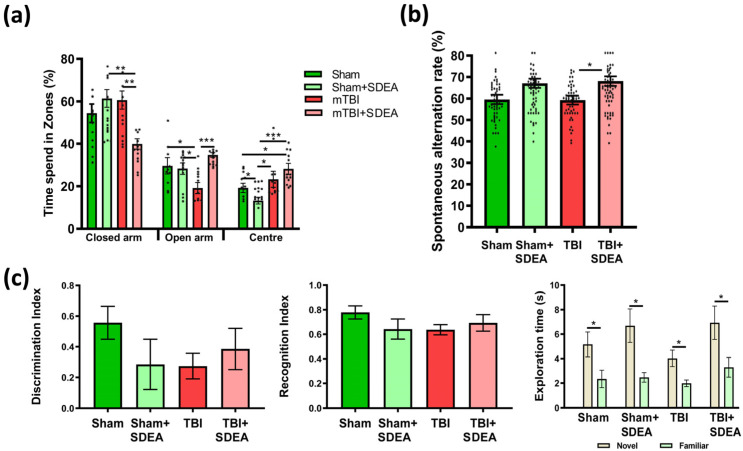
Effects of mild traumatic brain injury (mTBI) and SDEA treatment on anxiety-like behavior, working memory, and recognition memory in mice. (**a**) Elevated Plus Maze. The percentage of time spent in the closed arms, open arms, and center of the maze is shown for the four experimental groups: “Sham”, “Sham + SDEA”, “mTBI”, and “mTBI + SDEA” (*n* = 14 per group). Individual data points are overlaid on the box plot. Data are expressed as mean ± SEM (%). Two-way ANOVA. Post hoc Tukey test: * *p* < 0.05, ** *p* < 0.01, *** *p* < 0.001. (**b**) Y-maze spontaneous alternation test. The percentage of spontaneous alternations (%). Because Y-maze alternation was measured across multiple sessions (4 trials × 14 animals), *n* was 56 per group. Values are shown as mean ± SEM. Two-way ANOVA. Post hoc Tukey test: * *p* < 0.05. (**c**) Novel Object Recognition (NOR) test. The graphs show the Discrimination Index, the Recognition Index, and the exploration time (s) for novel and familiar objects in the four groups. Within-group comparisons revealed significant differences between novel and familiar object exploration in all experimental groups (* *p* < 0.05), *n* = 14 per group. Data are expressed as mean ± SEM (%). Two-way ANOVA. Post hoc Tukey test.

Given that mild traumatic brain injury often produces subtle behavioral alterations without overt cognitive deficits, we first examined anxiety-related behavior to establish functional consequences of injury and treatment. The effects of mTBI and treatment with SDEA on anxiety-related behavior were assessed in the elevated plus maze (EPM) [22] (Figure 1a). A two-way ANOVA was performed separately for the closed arms, open arms, and central zone.

Closed Arms

The percentage of time spent in the closed arms did not significantly differ between groups “Sham” (54.42 ± 4.58%) and “mTBI” (60.61 ± 4.40%) groups, indicating that mTBI alone did not substantially alter avoidance behavior (F_1, 52_ = 3.89, *p* = 0.054). SDEA administration produced a statistically significant decrease in closed-arm occupancy in injured animals (“mTBI”: 60.61 ± 4.40% vs. “mTBI + SDEA”: 39.89 ± 2.56%, *p* = 0.003). Although the main effect of treatment did not reach statistical significance (F_1, 52_ = 3.26, *p* = 0.078), a strong interaction was detected (F_1, 52_ = 12.77, *p* = 0.0008), indicating that TBI-induced behavioral deficits were restored in SDEA-treated animals.

Open Arms

mTBI animals exhibited a clear behavioral tendency toward reduced open-arm exploration compared with sham controls (“Sham”: 29.63 ± 3.95%; “mTBI”: 19.13 ± 2.73%). SDEA treatment increased open-arm time in mTBI animals (“mTBI + SDEA”: 34.66 ± 1.15%), restoring exploration after trauma. Two-way ANOVA revealed a significant main effect of treatment (F_1, 52_ = 7.30, *p* = 0.0096) and interaction between trauma and treatment (F_1, 52_ = 10.06, *p* = 0.0027). These results demonstrate that SDEA exerts an anxiolytic-like effect, counteracting the trauma-induced reduction in open-arm exploration.

Central Zone

In the central zone, mTBI animals did not differ significantly from sham controls, indicating that traumatic injury alone did not alter exploratory behavior in this area. The mean percentage of time spent in the center was 19.21 ± 2.23% in Sham and 23.31 ± 3.81% in mTBI animals (*p* > 0.05). SDEA exerted bidirectional, trauma-dependent effects: it slightly reduced central exploration in sham mice (“Sham + SDEA”: 13.24 ± 1.59%) but increased it in injured animals (“mTBI + SDEA”: 28.21 ± 2.62%) compared to controls. Two-way ANOVA confirmed a significant main effect of trauma (F_1, 52_ = 12.94, *p* = 0.001) and a significant interaction between trauma and treatment (F_1, 52_ = 4.21, *p* = 0.0458), while the main effect of treatment alone was not significant (F_1, 52_ = 0.036, *p* = 0.85). These data suggest that SDEA selectively increased central-zone exploration in injured animals while slightly suppressing it in intact mice, indicating a state-dependent, bidirectional modulation of risk-assessment behavior rather than a uniform anxiolytic action.

Together, these findings demonstrate that mTBI induces an anxiety-like phenotype, characterized by reduced exploration of open zones. SDEA treatment attenuated these alterations, restoring the balance between risk-taking and avoidance behavior. This normalization of exploratory activity supports the neuroprotective and anxiolytic potential of SDEA following mild traumatic brain injury.

#### 2.1.2. Y-Maze

The Y-maze spontaneous alternation test measures working memory and exploratory strategy, relying on the mouse’s innate tendency to alternate between arms [23] (Figure 1b). It is a low-stress task sensitive to hippocampal-prefrontal coordination and well suited for mild TBI models, which may show subtle network inefficiency rather than overt cognitive loss.

mTBI did not significantly affect spontaneous alternation performance, whereas SDEA treatment significantly increased alternation rate in injured animals. Mean values were 59.56 ± 2.15% for “Sham”, 66.27 ± 2.28% for “Sham-SDEA”, 59.28 ± 2.01% “mTBI”, and 67.30 ± 2.35% “mTBI-SDEA”. Thus, SDEA improved working-memory-related behavior independent of trauma status (F_1, 220_) = 11.12, *p* = 0.001.

These findings show that mild injury alone did not impair working memory, consistent with literature reporting preserved Y-maze alternation after mTBI, while SDEA enhanced hippocampal network efficiency.

#### 2.1.3. Novel Object Recognition

The novel object recognition (NOR) test was employed to assess long-term recognition memory, a form of recognition-based memory that depends on hippocampal-cortical interactions, including the perirhinal, entorhinal, and hippocampal regions [24] (Figure 1c).

Two-way ANOVA revealed no significant main effects of Trauma (F_1, 52_ = 0.52, *p* = 0.47) or Treatment (F_1, 52_ = 0.40, *p* = 0.53), and no factors’ interaction (F_1, 52_ = 2.32, *p* = 0.13) for either the discrimination or recognition indices. Likewise, exploration times for the familiar and novel objects did not differ significantly between groups (*p* > 0.22). Within-group analysis demonstrated that mice in all experimental groups spent significantly more time exploring the novel object compared to the familiar one (*p* < 0.05), indicating preserved object recognition performance across groups.

The absence of changes in NOR performance indicates that recognition memory was largely unaffected by mild traumatic brain injury in this model. These findings highlight a dissociation between affective and cognitive outcomes of mTBI: while emotional regulation and synaptic plasticity are selectively vulnerable, baseline recognition memory and neuronal differentiation are resilient.

As expected for a mild TBI model, behavioral alterations were subtle and did not manifest as severe deficits across all parameters.

### 2.2. Effect of mTBI and SDEA Treatment on Hippocampal Neurogenesis

Because behavioral changes may reflect underlying alterations in hippocampal cellular plasticity, we next examined markers of adult neurogenesis in the dentate gyrus.

**Figure 2:**
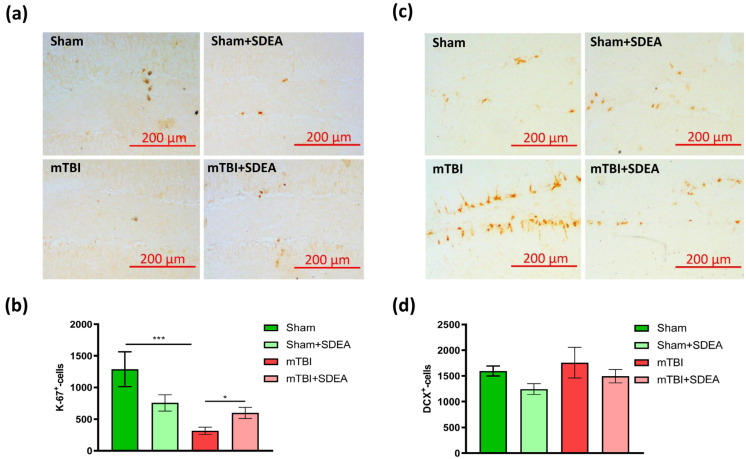
Immunohistochemical analysis of hippocampal neurogenesis markers Ki-67 and DCX after mTBI and SDEA treatment. (**a**,**b**) Ki-67 immunoreactivity in the dentate gyrus. Representative micrographs (**a**) show Ki-67^+^ proliferating cells (brown nuclei) in the subgranular zone of the dentate gyrus. Scale bar = 200 µm. Quantitative analysis (**b**) shows the number of Ki-67^+^ cells/mm^3^ (*n* = 25 slices per group). Data are expressed as mean ± SEM. Two-way ANOVA, Tukey’s post hoc test (* *p* < 0.05, *** *p* < 0.001). (**c**,**d**) DCX immunoreactivity in the dentate gyrus. Representative images (**c**) show DCX^+^ immature neurons in the subgranular zone of the dentate gyrus. Scale bar = 200 µm. Quantification (**d**) shows the number of DCX^+^ cells/mm^3^ (*n* = 25 slices per group). Data are expressed as mean ± SEM. Two-way ANOVA, Tukey’s post hoc test.

Ki-67-positive proliferating cells (Figure 2a) and DCX-positive cells (Figure 2b), representing immature neuronal populations, were analyzed within the SGZ of the DG, a region associated with adult hippocampal neurogenesis. Ki-67 is a marker of proliferating cells across all active phases of the cell cycle, whereas DCX labels immature neurons and reflects early neuronal differentiation within the adult neurogenic niche [25,26]. These markers allow phase-specific assessment of the neurogenic process.

For ki-67, two-way ANOVA revealed a strong main effect of trauma (F_1, 96_ = 12.35, *p* = 0.0007) and a significant interaction between trauma and treatment (F_1, 96_ = 6.41, *p* = 0.013). mTBI markedly reduced the number of Ki-67-positive cells compared with sham controls (“mTBI”: 317.3 ± 57.0 vs. “Sham”: 1288.4 ± 275.5, cells/mm^3^, mean ± SEM), indicating post-traumatic suppression of cell proliferation in the subgranular zone (SGZ). SDEA treatment partially restored proliferative activity in injured mice (599.5 ± 87.5 cells/mm^3^), while not affecting baseline proliferation in sham animals (757.1 ± 127.8 cells/mm^3^). Tukey’s post hoc test confirmed a significant difference between “Sham” and “mTBI” (*p* = 0.0003) and between “Sham” and “mTBI + SDEA” (*p* = 0.0163) (Figure 2c). These data demonstrate that mTBI strongly inhibits hippocampal cell proliferation, whereas SDEA exerts a restorative effect, counteracting trauma-induced reduction in Ki-67 expression and suggesting protection of the neurogenic niche.

Analysis of DCX-positive cells revealed a different pattern. Two-way ANOVA showed no significant main effect of trauma (F_1, 96_ = 1.34, *p* = 0.25) and no factors’ interaction (F_1, 96_ = 0.06, *p* = 0.80), though there was a near-significant trend for a treatment effect (F_1, 96_ = 2.97, *p* = 0.088). Post hoc analysis did not reveal statistically significant group differences (*p* > 0.05) (Figure 2d). Thus, DCX marks late progenitors and immature neurons, which remained stable across groups, despite the pronounced reduction in Ki-67-positive proliferating cells.

The divergent responses of Ki-67 and DCX indicate a phase-dependent modulation of hippocampal neurogenesis following mTBI. Because the assessment was performed 11 weeks after injury, the acute inflammatory effects of mTBI likely exerted their strongest impact on the early proliferative phase, reducing Ki-67^+^ cell numbers, whereas the pool of DCX^+^ immature neurons, which reflects a later, post-mitotic stage of neurogenesis, appears largely preserved at this chronic time point. This dissociation likely reflects the temporal delay between cell proliferation and neuronal maturation [25,26]. Together, these results support a phasic model in which mTBI transiently disrupts the earliest stage of neurogenesis, whereas SDEA may exert stage-specific effects by partially restoring proliferation while preserving immature neuron numbers required for hippocampal plasticity and cognitive recovery.

### 2.3. Effect of mTBI and SDEA Treatment on Arc Production

Beyond cell proliferation and differentiation, successful neurogenesis requires the functional integration of newborn neurons into existing hippocampal networks. Although functional integration of newborn neurons is a component of adult neurogenesis, immediate-early gene Arc (activity-regulated cytoskeleton-associated protein) expression was assessed here as a general marker of activity-dependent plasticity within the dentate gyrus, without implying cell-type specificity. This integration depends on activity-regulated synaptic mechanisms that enable newly formed granule cells to participate in memory encoding [27,28]. Therefore, after characterizing proliferation and maturation markers (Ki-67, DCX), we next evaluated Arc expression in the dentate gyrus to assess whether the observed changes in neurogenesis are accompanied by alterations in activity-dependent plasticity.

**Figure 3:**
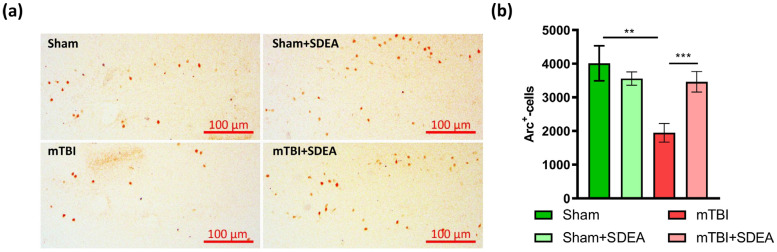
Immunohistochemical detection of Arc^+^ cells in the dentate gyrus after mTBI and SDEA treatment. (**a**) Representative micrographs showing Arc^+^-immunopositive cells (brown nuclei) in the DG. Scale bar = 100 µm. (**b**) Quantification of Arc^+^ cells/mm^3^ (*n* = 25 slices per group). Data are shown as mean ± SEM (cells/mm^3^). Two-way ANOVA, Tukey’s post hoc test (** *p* < 0.01, *** *p* < 0.001).

We measured the number of Arc-positive cells in the dentate gyrus across four groups (Figure 3a). After mTBI, Arc expression markedly decreased compared to sham animals (1948.8 ± 294.4 vs. 4009.8 ± 521.4, cells/mm^3^, *p* = 0.0004). Treatment with SDEA restored Arc levels in the injured group (3219.1 ± 345.0 cells/mm^3^), which were significantly higher than in “mTBI” mice (*p* = 0.0078) and became comparable to sham controls (*p* = 0.400). In contrast, SDEA had little effect in sham animals (3558.6 ± 199.4 vs. 4009.8 ± 521.4, cells/mm^3^, *p* = 0.426). Overall, these findings show that mTBI strongly suppresses Arc expression in the DG, and SDEA selectively restores Arc after injury, without changing baseline Arc levels in uninjured mice. The two-way ANOVA revealed a significant main effect of Trauma (F_1, 96_ =11.69, *p* = 0.00095), no significant main effect of Treatment (F_1, 96_ =1.12, *p* = 0.293), and a significant factors’ interaction (F_1, 96_ =5.96, *p* = 0.0166) (Figure 3b).

These findings show that mTBI strongly suppresses Arc expression, consistent with evidence that reduced Arc reflects weakened excitatory drive and impaired experience-dependent synaptic remodeling [27]. SDEA selectively reversed this deficit in injured animals, suggesting that it enhances synaptic activation and promotes re-engagement of dentate granule cells in hippocampal processing. The pattern in which Arc expression decreases in parallel with reduced proliferation (Ki-67) and recovers after SDEA is consistent with proposals that Arc interacts with activity-dependent processes that may indirectly influence neurogenic integration [28]. Together, these results indicate that SDEA restores activity-dependent signaling pathways that are crucial for functional recruitment of hippocampal circuits after mild brain injury.

### 2.4. Spine Remodeling After mTBI and SDEA Treatment

Since neurogenic and synaptic processes are tightly coupled within hippocampal networks, we subsequently assessed dendritic spine architecture and synaptic protein expression. Dendritic spine morphology reflects the structural basis of synaptic plasticity and functional connectivity in the hippocampus. Mature mushroom spines are associated with stable, high-conductance excitatory synapses, whereas thin spines and filopodia represent dynamic, plastic structures involved in learning-related remodeling [29,30]. mTBI often disrupts this balance, reducing mature synapses and promoting unstable spine types, which contribute to long-term synaptic inefficiency. To assess whether SDEA treatment could counteract these chronic morphological alterations, we analyzed dendritic spine density in hippocampal neurons 11 weeks after injury.

#### 2.4.1. CA1 Apical Dendrites

**Figure 4:**
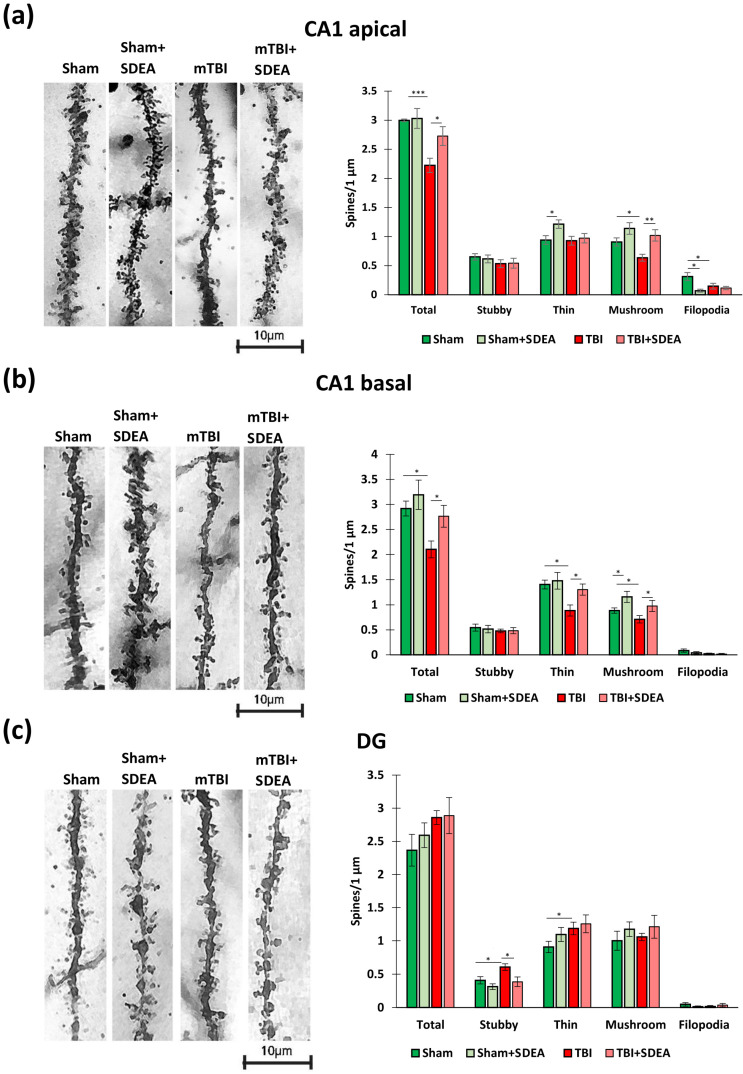
Dendritic spine morphology in apical and basal dendrites of CA1 pyramidal neurons and dendrites of dentate gyrus granule cells after mTBI and SDEA treatment. (**a**) CA1 apical dendrites. Representative Golgi-stained apical dendritic segments from the four experimental groups (**left**). Scale bar = 10 µm. Quantification of total spine density and spine subtypes (stubby, thin, mushroom, filopodia) is shown as spines/µm (*n* = 15 dendrites per group from 3 mice) (**right**). Data are expressed as mean ± SEM. Two-way ANOVA, Tukey’s post hoc test (* *p* < 0.05, ** *p* < 0.01, *** *p* < 0.001). (**b**) CA1 basal dendrites. Representative Golgi-stained basal dendritic segments from the four experimental groups (**left**). Scale bar = 10 µm. Quantification of total spine density and spine subtypes (stubby, thin, mushroom, filopodia) is shown as spines/µm (*n* = 15 dendrites per group from 3 mice) (**right**). Data are expressed as mean ± SEM. Two-way ANOVA, Tukey’s post hoc test (* *p* < 0.05). (**c**) Dentate gyrus (DG) dendrites. Representative Golgi-stained dendritic segments from DG granule neurons (**left**). Scale bar = 10 µm. Quantification of total spine density and spine subtypes (stubby, thin, mushroom, filopodia) is shown as spines/µm (*n* = 15 dendrites per group from 3 mice) (**right**). Data are expressed as mean ± SEM. Two-way ANOVA, Tukey’s post hoc test (* *p* < 0.05).

Two-way ANOVA revealed that both trauma and treatment significantly affected the total density of dendritic spines in CA1 apical dendrites (Trauma: F_1, 56_ = 12.45, *p* = 0.0010; Treatment: F_1, 56_ = 5.60, *p* = 0.0225), with no interaction between the factors (*p* = 0.39). TBI significantly reduced total spine density (“Sham”: 2.99 ± 0.09 vs. “mTBI”: 2.2 ± 0.12 spines/µm; *p* = 0.004), whereas treatment with SDEA partially restored it (“mTBI + SDEA”: 2.72 ± 0.15; *p* = 0.047 vs. “mTBI”) reaching levels not different from controls (*p* = 0.61) (Figure 4a).

For mushroom dendritic spines, both trauma (F_1, 56_ = 5.90, *p* = 0.019) and treatment (F_1, 56_ = 14.74, *p* = 0.0004) had strong effects. Mushroom spine density dropped after trauma (“Sham”: 0.91 ± 0.07 vs. “mTBI”: 0.64 ± 0.06; spines/µm, *p* = 0.003) and significantly increased under SDEA (“TBI + SDEA”: 1.02 ± 0.09; *p* = 0.012 vs. “mTBI”), suggesting partial restoration of mature synapses.

For thin dendritic spines, treatment exerted a significant effect (F_1, 56_ = 4.85, *p* = 0.033). SDEA elevated thin spine density in predominantly in sham animals (“Sham + SDEA”: 1.21 ± 0.07 vs. “Sham”: 0.94 ± 0.07; spines/µm, *p* = 0.041). These changes may reflect enhanced structural plasticity and turnover of excitatory synapses.

For stubby dendritic spines, no significant effects of trauma or treatment were found. The mean values remained stable (0.53–0.65 spines/µm), indicating that transitional spine forms are relatively resistant to both injury and pharmacological modulation.

For filopodia dendritic spines, a significant treatment effect (F_1, 56_ = 9.88, *p* = 0.003) and Trauma × Treatment interaction (F_1, 56_ = 5.82, *p* = 0.020) were observed. Filopodia density was highest in “Sham” (0.31 ± 0.06, spines/µm) and decreased under SDEA (0.07 ± 0.02, spines/µm; *p* = 0.001). This reduction may reflect stabilization of dendritic contacts and suppression of excessive spine turnover. Thus, mTBI caused a significant loss of mature mushroom spines and reduced overall spine density, while SDEA reversed these deficits and limited immature protrusions, suggesting structural stabilization and synaptic recovery in CA1 apical dendrites.

#### 2.4.2. CA1 Basal Dendrites

In CA1 basal dendrites, two-way ANOVA revealed significant main effects of both trauma and treatment on the total dendritic spine density in CA1 basal dendrites (Trauma: F_1, 56_ = 9.36, *p* = 0.0042; Treatment: F_1, 56_ = 5.27, *p* = 0.0276), with no significant interaction (*p* = 0.35). Following trauma, total spine density decreased from 2.92 ± 0.14 spines/µm in “Sham” to 2.10 ± 0.16 spines/µm in “mTBI” (*p* = 0.006). SDEA administration partially restored spine density to 2.76 ± 0.21 spines/µm (*p* = 0.041 vs. “mTBI”), approaching control levels (“Sham + SDEA”: 3.19 ± 0.28) (Figure 4b).

For mushroom dendritic spines, significant main effects of both trauma (F_1, 56_ = 4.28, *p* = 0.046) and treatment (F_1, 56_ = 9.83, *p* = 0.0034) were detected. Mushroom spine density dropped after trauma (“mTBI”: 0.710 ± 0.069 spines/µm vs. “Sham”: 0.885 ± 0.051 spines/µm; *p* = 0.028), while SDEA increased this parameter both in injured (“mTBI + SDEA”: 0.976 ± 0.105 spines/µm; *p* = 0.039 vs. “mTBI”) and sham animals (“Sham + SDEA”: 1.158 ± 0.105 spines/µm; *p* = 0.021 vs. “Sham”). These results indicate that SDEA enhances maintenance and restoration of mature, mushroom-type synapses.

Both trauma (F_1, 56_ = 8.95, *p* = 0.0050) and treatment (F_1, 56_ = 4.52, *p* = 0.0404) significantly affected thin spines. Trauma reduced their density from 1.40 ± 0.08 to 0.89 ± 0.10 spines/µm (*p* = 0.008), while treatment promoted partial recovery (“mTBI + SDEA”: 1.30 ± 0.10; *p* = 0.041 vs. “mTBI”). Thin spines, being structurally plastic, appear highly responsive to both injury and pharmacological stimulation.

Neither trauma nor treatment significantly influenced stubby spine density (Trauma: F_1, 56_ = 0.68, *p* = 0.42; Treatment: F_1, 56_ = 0.04, *p* = 0.84). Mean values remained within a narrow range (0.47–0.54 spines/µm) across all groups, indicating relative structural stability of this intermediate spine form under both conditions.

Filopodia density was significantly affected by trauma (F_1, 56_ = 4.87, *p* = 0.034) but not by treatment (*p* = 0.24). Post hoc tests showed a reduction in “mTBI” animals (0.020 ± 0.014) compared to “Sham” (0.085 ± 0.031; *p* = 0.044), consistent with trauma-induced suppression of nascent spine formation. Treatment did not further alter this parameter (“mTBI + SDEA”: 0.014 ± 0.014; *p* = 0.61 vs. “mTBI”).

Thus, in basal dendrites of CA1 pyramidal neurons, mild traumatic brain injury caused a substantial reduction in total spine density, driven primarily by the loss of mushroom and thin spines. mSDEA treatment significantly mitigated these effects, restoring both mature (mushroom) and plastic (thin) spine populations. Stubby spines remained unaffected, while filopodia were reduced following trauma, suggesting suppressed synaptogenesis. Together, these data indicate that SDEA preserves dendritic integrity and promotes synaptic stability in basal dendrites after mTBI.

#### 2.4.3. Dentate Gyrus Granule Cells

In DG granule neurons, two-way ANOVA showed a trend of increased total spine density after trauma (F_1, 56_ = 3.90, *p* = 0.056), without significant treatment effects (*p* = 0.52). Trauma increased mean density from 2.37 ± 0.23 (“Sham”) to 2.86 ± 0.10 spines/µm (“TBI”; *p* = 0.059) insignificantly, consistent with reactive synaptogenesis in the DG (Figure 4c).

For mushroom dendritic spines, no significant differences were detected (*p* > 0.18). Densities remained stable across all groups (1.00–1.21 spines/µm), suggesting that mature synaptic contacts in the DG are less affected by trauma or treatment than in CA1.

Trauma significantly increased thin spine density (F_1, 56_ = 4.69, *p* = 0.037), whereas treatment had no independent effect (*p* = 0.21). TBI animals exhibited higher thin spine numbers (1.19 ± 0.09 vs. 0.91 ± 0.08 spines/µm; *p* = 0.049), and treatment further elevated them (1.26 ± 0.13 spines/µm; *p* = 0.041 vs. “TBI”). This enhancement may reflect adaptive plasticity and structural compensation in granule cells.

For stubby dendritic spines, both Trauma (F_1, 56_ = 6.62, *p* = 0.014) and Treatment (F_1,56_ = 8.88, *p* = 0.005) were significant. Stubby spine density rose after trauma (0.605 ± 0.047 vs. 0.407 ± 0.051 spines/µm; *p* = 0.013) and normalized with SDEA (0.386 ± 0.068 spines/µm; *p* = 0.031 vs. “TBI”), suggesting reduced structural immaturity.

For filopodia dendritic spines, no significant changes were found (*p* > 0.15). Filopodia remained rare (0.01–0.05 spines/µm), consistent with limited de novo spine formation in adult granule cells. Thus, DG neurons exhibited a trauma-induced increase in thin spines, coupled with elevated stubby forms, suggesting reactive plasticity. SDEA modulated this response by normalizing stubby spines, thus favoring a more flexible yet stable synaptic architecture.

### 2.5. SDEA Suppresses mTBI-Induced Astroglial Activation

**Figure 5:**
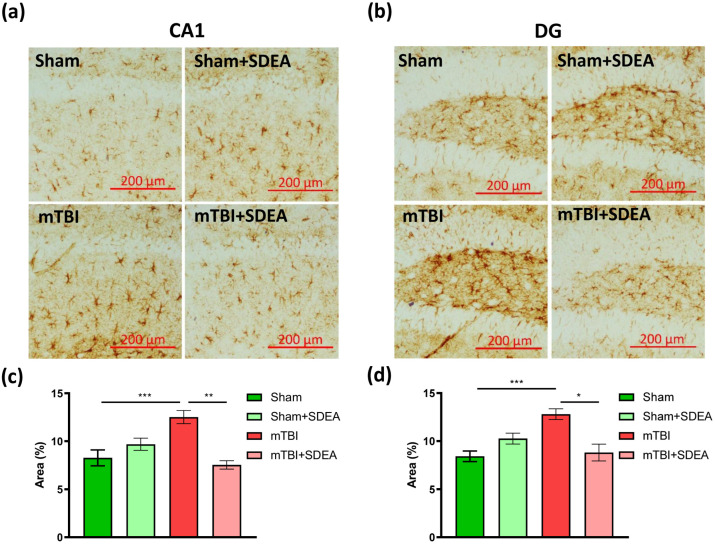
GFAP immunoreactivity in the CA1 region and dentate gyrus (DG) after mTBI and SDEA treatment. (**a**) Representative GFAP-stained astrocytes in the CA1 region of the hippocampus. Scale bar = 200 µm. (**b**) Representative GFAP immunostaining in the DG across the same experimental groups. Scale bar = 200 µm. (**c**) Quantification of GFAP-positive area (%) in CA1 (*n* = 25 slices per group). Data are presented as mean ± SEM. Two-way ANOVA. Tukey post hoc test (** *p* < 0.01, *** *p* < 0.001). (**d**) Quantification of GFAP-positive area (%) in DG (*n* = 25 slices per group). Data are presented as mean ± SEM. Two-way ANOVA. Tukey post hoc test (* *p* < 0.05, *** *p* < 0.001).

Astrocytic activation is a hallmark of post-traumatic neuroinflammation and is characterized by increased GFAP expression, cellular hypertrophy, and altered metabolic and glutamatergic support [31,32]. Persistent GFAP elevation reflects chronic reactivity and is widely used as a biomarker of astroglial injury in both tissue and serum studies. To determine whether SDEA treatment modulates astroglial activation in the hippocampus during the chronic phase after mild TBI, we quantified the GFAP-positive area in the DG (Figure 5a) and CA1 subregions (Figure 5b) 11 weeks post-injury.

Quantitative assessment of GFAP-positive area in the CA1 region demonstrated a significant effect of treatment (F_1, 96_ = 7.27, *p* = 0.0084) and a strong interaction between trauma and treatment (F_1, 96_ = 23.53, *p* < 0.00001), indicating that SDEA efficiently modulated astroglial activation following mild TBI. The main effect of trauma was not significant (*p* = 0.12). In the “mTBI” group, the GFAP-positive area was markedly elevated (12.52 ± 0.68%), reflecting persistent astrocytic reactivity 11 weeks post-injury. SDEA treatment significantly reduced this value to 7.53 ± 0.43%, restoring it close to control levels. In sham-operated animals, SDEA caused no significant change compared with vehicle (9.69 ± 0.63% vs. 8.26 ± 0.83%), confirming that its effect was specific to post-traumatic conditions (Figure 5c).

In the DG region, two-way ANOVA showed a significant main effect of trauma (F_1, 96_ = 5.06, *p* = 0.027), a tendency for treatment (*p* = 0.10), and a strong interaction between trauma and treatment (F_1, 96_ = 20.12, *p* < 0.0001), indicating that SDEA significantly modulated astroglial reactivity in injured animals. In the “mTBI” group, the GFAP-positive area was markedly elevated (12.81 ± 0.56%), indicating persistent astrocytic activation 11 weeks after injury. Treatment with SDEA reduced this value to 8.81 ± 0.87%, restoring it toward control levels. In sham-operated mice, SDEA produced no significant change compared with vehicle (10.27 ± 0.57% vs. 8.42 ± 0.55%), confirming that its effect was specific to post-traumatic conditions (Figure 5d).

These results indicate that SDEA effectively attenuates long-term astroglial activation across the hippocampus, thereby supporting recovery of glia–neuron homeostasis and potentially facilitating synaptic and neurogenic repair after mild traumatic brain injury.

### 2.6. SDEA Normalizes BIN1 Expression and Restores Synaptic Marker Balance After mTBI

The structural and functional integrity of synapses critically depends on the surrounding glial environment, particularly astrocytes, which form the “tripartite synapse” and regulate glutamate homeostasis, stabilization of postsynaptic structures, and AMPA-mediated transmission. In the preceding section, we showed that mTBI induces pronounced astrocytic activation and an increase in GFAP-positive area in both CA1 and DG, indicating reactive remodeling of the glial component of the hippocampal network. Such glial changes can directly influence synaptic protein composition, as reactive astrocytes disrupt glutamate uptake, release pro-inflammatory mediators, and alter local metabolic conditions, processes that collectively lead to reductions in key structural proteins (Synaptophysin, PSD-95) and disturbances in AMPA receptor subunit expression [33]. AMPA receptor subunits GluR1 and GluR2 determine synaptic strength and Ca^2+^ permeability, with GluR2 conferring Ca^2+^-impermeability and stabilizing mature synaptic contacts [34,35,36]. Disruption of these proteins after TBI reflects impaired structural maintenance of excitatory synapses. Therefore, the next step of our analysis was to quantify presynaptic (Synaptophysin) and postsynaptic density (PSD-95) proteins, as well as AMPA receptor subunits (GluR1/2), to determine how astrocytic reactivity in mTBI relates to alterations in synaptic architecture and excitatory transmission, and to assess the extent to which SDEA can restore these molecular indices.

#### 2.6.1. Synaptophysin

**Figure 6:**
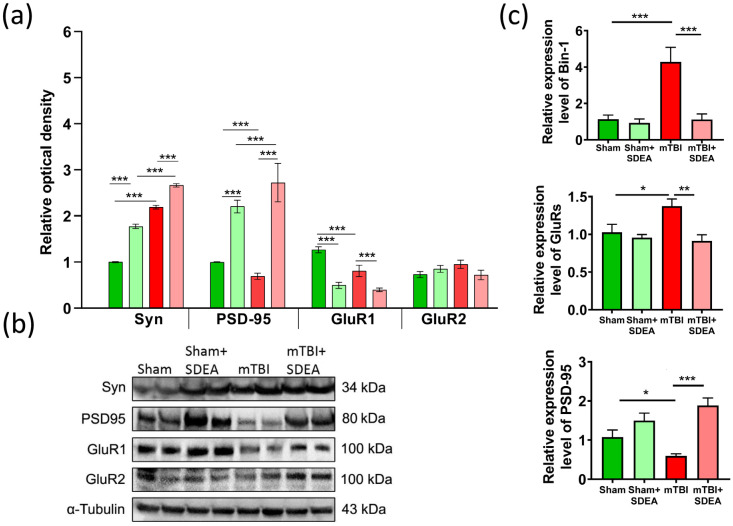
Expression of synaptic proteins and AMPA receptor subunits after mTBI and SDEA treatment. (**a**) Quantification of relative optical density of synaptic proteins Synaptophysin (Syn), PSD-95, and AMPA receptor subunits GluR1 and GluR2 across the four experimental groups. Data are shown as mean ± SEM (relative optical density, normalized to α-tubulin); *n* = 6 (animals per group). Two-way ANOVA. Tukey post hoc: *** *p* < 0.001. (**b**) Representative Western blot bands showing protein levels of Syn (~34 kDa), PSD-95 (~80 kDa), GluR1 (~100 kDa), GluR2 (~100 kDa), and loading control α-Tubulin (~43 kDa) from hippocampal homogenates of all experimental groups. (**c**) Relative mRNA expression of Bin1, GluRs (combined AMPA receptor subunits), and PSD-95 measured by qPCR. Values are presented as mean ± SEM (relative expression normalized to housekeeping genes); *n* = 3 (animals per group). Two-way ANOVA. Tukey post hoc: * *p* < 0.05, ** *p* < 0.01, *** *p* < 0.001.

Synaptophysin levels were significantly increased after mTBI compared to sham animals (“Sham”: 1.00 ± 0.01; “mTBI”: 2.14 ± 0.02, *p* < 0.001), indicating increased presynaptic vesicle density, potentially reflecting compensatory presynaptic remodeling. Importantly, SDEA treatment further increased synaptophysin expression in mTBI mice compared to untreated mTBI animals (*p* < 0.001), suggesting that SDEA potentiates injury-associated presynaptic remodeling (Figure 6a,b).

#### 2.6.2. PSD-95

mTBI animals exhibited a significant reduction in PSD-95 protein levels compared to sham controls (“Sham”: 1.00 ± 0.01; “mTBI”: 0.66 ± 0.01, *p* < 0.001). SDEA treatment significantly increased PSD-95 expression in mTBI mice compared to untreated mTBI animals (*p* < 0.001), indicating attenuation of trauma-associated postsynaptic alterations (Figure 6a,b).

#### 2.6.3. GluR1

mTBI was associated with a reduction in GluR1 protein expression relative to sham controls (“Sham”: 1.27 ± 0.03; “mTBI”: 0.81 ± 0.05, *p* < 0.001). SDEA treatment led to a further decrease in GluR1 levels compared to the “mTBI” group, suggesting additional modulation of AMPA receptor subunit composition during the post-injury period (Figure 6a,b). This additional reduction in GluR1 may reflect adaptive rebalancing of AMPA receptor composition rather than further synaptic impairment.

#### 2.6.4. GluR2

No significant differences in GluR2 protein levels were detected between “Sham”, “mTBI”, and “mTBI + SDEA” groups (Figure 6a,b), indicating that neither mild traumatic brain injury nor SDEA treatment significantly affected GluR2 expression at the protein level (Figure 6a,b).

Collectively, these data indicate that mTBI induces alterations in synaptic protein composition, including reduced PSD-95 and changes in AMPA receptor subunits. SDEA treatment attenuated several trauma-associated synaptic alterations, particularly at the level of postsynaptic scaffolding proteins and receptor composition, consistent with partial stabilization of excitatory synapses.

To evaluate transcriptional changes accompanying the synaptic protein alterations, we analyzed mRNA expression of Psd95, GluR subunits and Bin1 in the hippocampus by quantitative PCR. BIN1 (Bridging Integrator 1) protein was included in this study as a key regulator of membrane curvature and endocytic trafficking, processes that are crucial for maintaining synaptic structure and receptor dynamics. BIN1 has been repeatedly identified as one of the major genetic risk factors for Alzheimer’s disease, where its dysregulation contributes to abnormal membrane remodeling, Tau pathology, and synaptic loss [37,38,39]. Given the growing evidence that TBI accelerates neurodegenerative processes and shares common molecular pathways with Alzheimer’s disease, assessing BIN1 expression provides important insight into how mild TBI affects neuronal membrane homeostasis and synaptic stability. By analyzing BIN1 together with synaptic markers (Synaptophysin, PSD-95, GluR), we aimed to determine whether the observed synaptic alterations after mTBI are linked to BIN1-dependent mechanisms and whether SDEA treatment can normalize this Alzheimer-like molecular signature.

#### 2.6.5. Bin-1 mRNA

Two-way ANOVA revealed significant main effects of trauma (F_1, 8_ = 13.34, *p* = 0.0016), treatment (F_1, 8_ = 13.53, *p* = 0.0015), and a significant interaction (F_1, 8_ = 10.50, *p* = 0.0041). Bin-1 expression was markedly increased in “mTBI” animals (4.28 ± 0.80, SEM) compared to sham controls (1.13 ± 0.23, *p* < 0.001), while SDEA reduced Bin-1 mRNA to near-control levels (1.12 ± 0.31, *p* < 0.001 vs. “mTBI”) (Figure 6c).

#### 2.6.6. Psd-95 mRNA

A strong treatment effect (F_1, 8_ = 23.58, *p* = 0.0013) and a significant trauma × treatment interaction (F_1, 8_ = 6.03, *p* = 0.039) were observed. Psd-95 mRNA was significantly downregulated after mTBI (0.69 ± 0.12) compared to sham (1.14 ± 0.15, *p* = 0.002), while SDEA treatment significantly increased Psd-95 mRNA expression in mTBI mice compared to untreated mTBI animals (*p* < 0.001), indicating reversal of trauma-associated transcriptional downregulation (Figure 6c).

#### 2.6.7. GluR Subunits mRNA

Analysis of combined GluR1/2 transcripts showed significant effects of treatment (F_1, 8_ = 9.21, *p* = 0.0162) and interaction (F_1, 8_ = 5.01, *p* = 0.043). mTBI led to a decrease in AMPA receptor mRNA (0.79 ± 0.11 vs. sham 1.02 ± 0.08, *p* < 0.05), indicating impaired transcription of excitatory receptor subunits. SDEA partially restored GluR expression (0.96 ± 0.10, *p* < 0.05 vs. “mTBI”) (Figure 6c).

Thus, at the transcriptional level, mTBI induced a characteristic pattern of Bin-1/GluR upregulation and Psd95 downregulation, reflecting disturbed membrane trafficking and synaptic gene expression. The concordance between PSD-95 protein and mRNA levels contrasts with the lack of direct correspondence observed for AMPA receptor subunits. This dissociation likely reflects the distinct regulatory mechanisms governing these proteins. While PSD-95 expression is largely transcriptionally regulated, AMPA receptor abundance at synapses is dynamically controlled by activity-dependent trafficking, endocytosis, and recycling, which can uncouple protein levels from transcript abundance [33,34]. SDEA administration attenuated these trauma-associated transcriptional changes, consistent with modulation of synaptic gene expression programs following injury.

### 2.7. Serum Neuroglial Markers Following mTBI and SDEA Treatment

**Figure 7:**
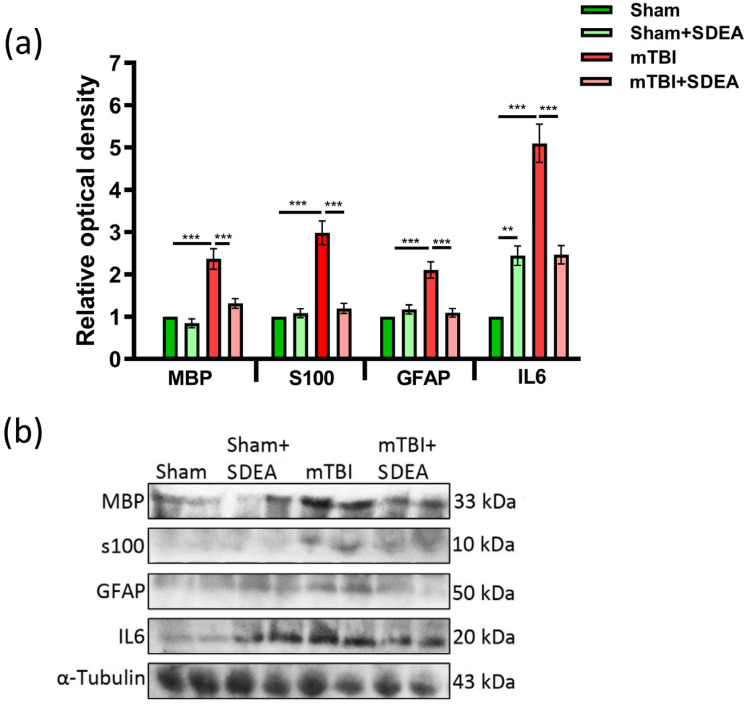
Serum levels of glial and inflammatory markers after mTBI and SDEA treatment. (**a**) Quantification of relative optical density of MBP, S100, GFAP, and IL-6 in serum samples from the four experimental groups. Data are presented as mean ± SEM (relative optical density normalized to α-tubulin); n = 6 (animals per group). Two-way ANOVA. Tukey post hoc test (** *p* < 0.01, *** *p* < 0.001). (**b**) Representative Western blot bands for MBP (~33 kDa), S100 (~10 kDa), GFAP (~50 kDa), IL-6 (~20 kDa), and loading control α-Tubulin (~43 kDa) from serum samples of the four experimental groups.

Serum levels of neuroglial proteins (MBP, S100B, GFAP) and cytokines (IL-6) are widely used biomarkers of CNS injury and systemic neuroglial stress [40,41,42]. Two-way ANOVA revealed significant main effects of both Trauma and Treatment, as well as factors’ interactions for all markers (*p* < 0.01). Serum levels of MBP (F_1, 20_ = 40.8, *p* < 0.0001), S100 (F_1, 20_ = 42.3, *p* < 0.0001), GFAP (F_1, 20_ = 17.8, *p* = 0.0004), and IL-6 (F_1, 20_ = 55.6, *p* < 0.0001) were all markedly increased in the “TBI” group compared to controls (all Tukey *p* < 0.001). SDEA treatment significantly attenuated these elevations, reducing MBP to 1.31 ± 0.11 vs. 2.36 ± 0.24 (*p* = 0.0002), S100 to 1.19 ± 0.12 vs. 2.98 ± 0.28 (*p* < 0.0001), GFAP to 1.09 ± 0.10 vs. 2.10 ± 0.19 (*p* < 0.0001), and IL-6 to 2.46 ± 0.22 vs. 5.10 ± 0.45 (*p* < 0.0001). No significant differences were observed between “Sham” and “Sham-SDEA” groups, indicating that SDEA alone did not alter baseline serum profiles (Figure 7a,b).

The coordinated increase in MBP, S100, GFAP, and IL-6 after mTBI reflects axonal/glial injury and systemic inflammation, consistent with increased neuroglial stress and systemic responses associated with CNS injury. SDEA treatment significantly reduced serum levels of all markers elevated by mTBI.

## 3. Discussion

Mild traumatic brain injury is increasingly recognized as a condition that can produce long-lasting glial reactivity and synaptic dysfunction even in the absence of overt neuronal loss or severe cognitive deficits [43,44,45,46]. In our study, mTBI elicited a coordinated set of molecular, structural, and mild behavioral changes in the hippocampus, including astrocytic activation, synaptic protein loss, dendritic spine alterations, reduced activity-dependent Arc expression, and impaired ki-67-positive proliferation within the DG SGZ. Importantly, treatment with stearidonic acid ethanolamide (SDEA) attenuated many of these alterations and ameliorated the anxiety-like behavior induced by injury. Given the mild nature of the injury, behavioral alterations were subtle, and SDEA effects should be interpreted as modulation of behavioral responses under injury conditions rather than full normalization of overt deficits.

A key finding of our study was the elevation of GFAP within hippocampal tissue, which indicates local reactive astrogliosis. Reactive astrocytes undergo hypertrophy, cytoskeletal remodeling, and altered regulation of glutamate and cytokine signaling [47,48,49,50], changes that can profoundly influence neuronal excitability and synaptic stability. In parallel, we observed increases in serum GFAP, S100B, MBP, and IL-6. These circulating proteins represent a distinct biological dimension: they reflect glial stress and axonal strain. Because blood–brain barrier integrity was not directly assessed in the present study, changes in circulating GFAP, S100B, MBP, and IL-6 should be interpreted as indirect indicators rather than definitive evidence of BBB disruption [51,52,53,54]. Thus, tissue and serum markers reflect complementary but different aspects of the post-traumatic response.

In line with this glial activation, we also detected coordinated alterations in synaptic structure and plasticity-related signaling. Synaptic alterations were heterogeneous, with decreases in PSD-95 and GluR1 but increases in synaptophysin. Although these measurements reflect signals averaged across subregions, the magnitude and pattern of the changes align with the dendritic spine deficits we quantified specifically in CA1. PSD-95 is essential for anchoring and stabilizing AMPA receptors at postsynaptic sites, and its loss is a hallmark of impaired synaptic plasticity [55,56]. AMPA receptor dynamics are further compromised by inflammatory cytokines such as IL-6 and TNF-α, which promote GluR1 internalization and reduce synaptic incorporation [57,58]. The reduction in GluR1 that we observed is therefore consistent with the inflammatory milieu of mTBI and with the GFAP-based evidence of astrocytic activation. Together, these findings suggest that synaptic weakening after injury arises from convergent astrocytic and neuronal mechanisms.

Morphological analyses further revealed a clear regional specificity: dendritic spine density was markedly reduced in CA1 pyramidal neurons, affecting both apical and basal dendrites. DG granule cells showed fewer structural alterations than CA1, suggesting partial resistance in this mild-injury model. This pattern is strongly supported by previous work demonstrating that CA1 neurons are disproportionately vulnerable to metabolic, inflammatory, and mechanical stress due to their high excitatory drive, dense NMDA receptor expression, and selective susceptibility to glutamate-mediated toxicity [59,60]. Numerous studies have shown that CA1 pyramidal neurons exhibit pronounced synaptic and structural deficits even after mild injuries [4]. In contrast, DG granule neurons exhibited only modest structural changes. This pattern is compatible with the notion that the dentate gyrus possesses a unique neurogenic niche and high structural plasticity, which may provide some buffering capacity against injury-induced loss [61,62]. At the same time, several studies have shown that, although DG neurons often survive after traumatic brain injury, their dendrites and synapses can still undergo substantial degeneration [63], indicating that such resilience is relative rather than absolute. In this context, our finding of preserved DG spine morphology but marked CA1 spine loss suggests that mild trauma in our model primarily destabilizes CA1 networks, while DG granule cells either remain structurally intact or are better compensated by ongoing neurogenesis.

This regional dissociation, marked spine loss in CA1 with relative preservation of DG morphology, suggests that not only structural connectivity but also activity-dependent plasticity may be differentially affected across hippocampal subfields. Consistent with this idea, we next examined the immediate early gene Arc, a key marker of experience- and activity-dependent synaptic plasticity in the dentate gyrus, to determine whether mTBI and SDEA modulate hippocampal network engagement at the cellular level. Arc, an immediate-early gene essential for synaptic plasticity and AMPA receptor trafficking [64], was reduced in the DG following mTBI. While Arc is classically described as an immediate early gene, long-lasting changes in its expression have been observed in conditions involving chronic synaptic remodeling and persistent network dysfunction. Therefore, the altered Arc levels detected at 11 weeks post-mTBI likely reflect sustained activity-dependent plasticity rather than acute gene induction. Reduced Arc is typically associated with impaired memory encoding and diminished hippocampal network engagement [65,66]. Yet, in contrast to what might be predicted from Arc downregulation alone, the NOR test revealed no deficits. This apparent discrepancy likely reflects the multilayered nature of DG function. However, recognition memory relies not only on Arc-mediated plasticity but also on intact structural connectivity and pattern separation processes [67]. Because DG dendritic spines and DCX-positive cells were largely preserved in our study, the reduction in Arc may represent a functional but subthreshold impairment, insufficient to compromise NOR performance. These findings support the notion that molecular changes may precede or occur without measurable behavioral deficits [68]. Interestingly, while Heim et al. reported cognitive impairments despite minimal detectable anatomical changes, our animals exhibited the opposite dissociation: pronounced molecular and synaptic abnormalities, particularly in CA1, without measurable deficits in recognition or working memory. This suggests an even more subtle form of “silent” hippocampal dysfunction, in which injury-induced alterations in Arc expression, synaptic proteins, and dendritic spine architecture are effectively compensated at the behavioral level, at least in tasks primarily relying on DG-dependent pattern separation.

By contrast, CA1-dependent functions appear much more sensitive to the structural and molecular disruptions we observed. CA1 plays a central role in emotional regulation and stress reactivity [69,70], and CA1-selective synaptic weakening can increase anxiety-like behavior even in the absence of cognitive decline [59,71,72]. This matches our behavioral results: anxiety-like behavior increased after mTBI, whereas NOR and Y-maze performance remained intact. The preserved architecture of the DG together with only mild Arc and Ki-67 suppression suggests that DG networks could compensate for injury-related changes, supporting normal recognition memory performance. At the same time, the combination of CA1 spine loss, reduced PSD-95/GluR1, and strong astrocytic activation offers a clear mechanism for the heightened anxiety-like behavior.

Finally, the restorative actions of SDEA align with the broader biology of ω-3 polyunsaturated fatty acid-derived ethanolamides, which function as lipid mediators coordinating glial reactivity and synaptic stability. Synaptamide and related ethanolamides such as EPEA engage GPR110-dependent cAMP/PKA signaling, attenuate NF-κB-driven astrocytic activation, and support neurite and axonal growth [15,16,73,74]. Because astrocytic NF-κB activation contributes substantially to excitatory synapse vulnerability after mTBI [5,9], the reduction in GFAP-associated reactivity observed after SDEA likely reflects improved glial-synaptic homeostasis. ω-3-derived ethanolamides may also influence activity-dependent plasticity through their capacity to reduce neuroinflammatory signaling and thereby preserve the molecular environment required for experience-driven synaptic responses. Synaptamide has been shown to attenuate microglial activation and improve hippocampal plasticity in inflammatory conditions [75], suggesting that ω-3 ethanolamides can indirectly support plasticity-related gene expression by stabilizing glial-synaptic interactions. This is relevant given the central role of Arc in structural and functional synaptic adaptations [76]. Recent work demonstrates that astrocyte-dependent glutamate clearance regulates Arc translation and supports hippocampal spatial memory [77], illustrating how glial metabolic signaling can shape Arc-mediated plasticity. In this context, the normalization of Arc expression following SDEA treatment is consistent with a broader framework in which ω-3 ethanolamides restore the permissive environment for experience-dependent plasticity by dampening glial cytokine output and maintaining synaptic homeostasis. In parallel, several N-acylethanolamides including synaptamide, EPEA, and PEA are known to influence neurogenic pathways by reducing inflammatory stress and supporting the cellular environment required for progenitor proliferation. PEA has been shown to restore hippocampal neurogenesis under inflammatory and neuropathic conditions by attenuating glial activation [78,79], while DHA and its derivatives, including synaptamide, promote neuronal differentiation and neuritogenesis through GPR110-mediated cAMP/PKA signaling [20,80,81]. These mechanisms provide a plausible basis for the increase in Ki-67-positive cells observed after SDEA treatment, suggesting improved DG niche conditions through combined anti-inflammatory and pro-neurogenic actions. These convergent mechanisms provide a cohesive explanation for the observed normalization of synaptic protein expression, restoration of Arc-dependent plasticity, rescue of CA1 dendritic spines, and stimulation of Ki-67-positive proliferative activity. The accompanying reduction in anxiety-like behavior is consistent with the particular sensitivity of CA1 circuits to glial dysregulation and excitatory synaptic loss, supporting the interpretation that SDEA acts as a multi-target modulator capable of re-establishing hippocampal circuit stability after mTBI.

The observation that SDEA exerted both injury-independent effects and injury × treatment interactions suggests the involvement of multiple, context-dependent mechanisms of action. Some effects likely reflect baseline modulatory properties of SDEA on glial reactivity and synaptic organization under physiological conditions, whereas other effects become evident only in the presence of trauma-induced network dysfunction. Such dual action is consistent with a compound that both stabilizes homeostatic processes and counteracts pathological alterations triggered by mTBI.

In summary, our findings demonstrate that mTBI induces coordinated glial, synaptic, and neurogenic disturbances that are anatomically and functionally selective, disproportionately affecting CA1 while sparing DG-dependent recognition memory. The partial mismatch between Arc downregulation in DG and preserved NOR performance likely reflects subthreshold functional impairment insufficient to disrupt behavior. SDEA effectively mitigated astrocytic activation, preserved synaptic structure, restored molecular plasticity markers, and reduced anxiety-like behavior, highlighting its therapeutic potential as a multi-target modulator of post-traumatic hippocampal plasticity.

## 4. Materials and Methods

### 4.1. Isolation and Characterization of Stearidonic Acid Ethanolamide (SDEA)

The stearidonic acid ethanolamide (N-stearidonylethanolamine) used in this study was produced from lipid extracts of *Sardinops melanostictus*. To obtain the target N-acylethanolamide, polyunsaturated fatty acids were first converted to their ethyl ester forms, after which the esterified fraction was reacted with ethanolamine to generate PUFA ethanolamides. Following synthesis, the reaction products were extracted with chloroform and washed repeatedly with water to remove polar contaminants.

The crude mixture of ethanolamides was initially purified by silica gel column chromatography. Subsequent separation of individual PUFA ethanolamides was achieved by preparative HPLC using a Shimadzu LC-8A system equipped with a UV/VIS SPD-20A detector (λ = 205 nm) (Shimadzu, Kyoto, Japan). Chromatographic separation was performed on a Supelco Discovery HS C18 column (250 × 50 mm, 10 µm particle size) (Supelco, Bellefonte, PA, USA) under isocratic elution with ethanol/water (70:30, *v*/*v*) at a flow rate of 50 mL/min. Fractions containing N-acylethanolamides were collected, evaporated under reduced pressure, and subjected to GC and GC–MS analysis for final identification. The obtained SDEA was a pale yellow, slightly viscous oil with a purity of 94.8%. To determine the composition of the ethanolamide fraction, samples were derivatized to their trimethylsilyl (TMS) forms. Briefly, 1 mg of fatty acid ethanolamides was mixed with 50 µL of BSTFA (N,O-bis(trimethylsilyl)trifluoroacetamide) and incubated at 60 °C for 1 h under an argon atmosphere. After cooling, 1 mL of hexane was added, and 1 µL of the silylated solution was injected into a Shimadzu GC-2010 Plus system equipped with a flame ionization detector (Shimadzu, Kyoto, Japan) and a Supelco SLB™-5 ms capillary column (30 m × 0.25 mm) (Supelco, Bellefonte, PA, USA).

Gas chromatographic analysis was performed using the following temperature program: initial temperature 180 °C; ramp 2 °C/min to 260 °C; hold at 260 °C for 35 min. Injector and detector temperatures were both set to 260 °C. Structural confirmation of TMS-NAE derivatives was carried out by GC–MS using a Shimadzu TQ-8040 mass spectrometer (Shimadzu, Kyoto, Japan) with electron impact ionization (70 eV) and the same chromatographic conditions. Mass spectral analysis yielded a molecular ion at m/z 391 for TMS-N-SDEA, with a prominent fragment at m/z 376 corresponding to loss of a methyl group. Total ion chromatograms and representative mass spectra are shown in Supplementary Figure S1a,b.

### 4.2. Animals

Male C57BL/6J mice (8–10 weeks old) were bred and housed in the animal facility (vivarium) of the A.V. Zhirmunsky National Scientific Center of Marine Biology, Far Eastern Branch of the Russian Academy of Sciences (Vladivostok, Russia). All experimental groups were derived from the same local breeding colony, ensuring internal genetic consistency across comparisons. Adult male mice (3 months old; 28–30 g) were used in all experiments. Animals were housed in groups of four under standard laboratory conditions (22 ± 1 °C; 12:12 light/dark cycle; food and water ad libitum). Only male mice were used in this study to reduce biological variability associated with estrous cycle-dependent hormonal fluctuations and because the work was designed as an exploratory mechanistic analysis. A total of 68 mice were used (*n* = 17 per group: “Sham”, “Sham + SDEA”, “mTBI”, “mTBI + SDEA”). All procedures adhered to the EU Directive 2010/63/EU [82] and were approved by the Animal Ethics Committee of the National Scientific Center for Marine Biology, Far East Branch of the Russian Academy of Sciences (No. 2/2025, 10 February 2025).

### 4.3. mTBI Model

Mild traumatic brain injury was modeled using a standardized closed-head impact procedure. Mice were first placed in an induction chamber and anesthetized with 4.5% isoflurane (Laboratories Karizoo, SA, Barcelona, Spain) delivered in 100% oxygen using a rodent anesthetic vaporizer (VetFloTM, Kent Scientific Corporation, Torrington, CT, USA). After 1–3 min, when a stable level of anesthesia was confirmed, the scalp was shaved and a thin metallic head shield (1.25 mm) was fixed over the parietal area to distribute the mechanical load during impact.

For injury induction, animals were positioned on a flexible polyurethane support mounted on the platform of a vertical impact apparatus (IMP-1020, Shanghai TOW Intelligent Technology, Shanghai, China). The head shield was centered beneath the guide tube to ensure reproducibility of the impact coordinates. A 100 g weight was then elevated to 40 cm and released through the tube by an electromagnet, producing a controlled mild impact to the skull without craniotomy.

Immediately after the procedure, animals were examined for acute adverse events such as convulsions or external cranial deformation; neither was detected in any experimental group. Mice were then transferred to a warmed recovery surface and monitored until spontaneous movement, normal posture, and thermoregulation were restored. No mortality occurred throughout the study. Sham-operated animals underwent the same preparation steps, including anesthesia and placement under the device, but the weight was not released.

### 4.4. SDEA Preparation and Administration

**Figure 8:**
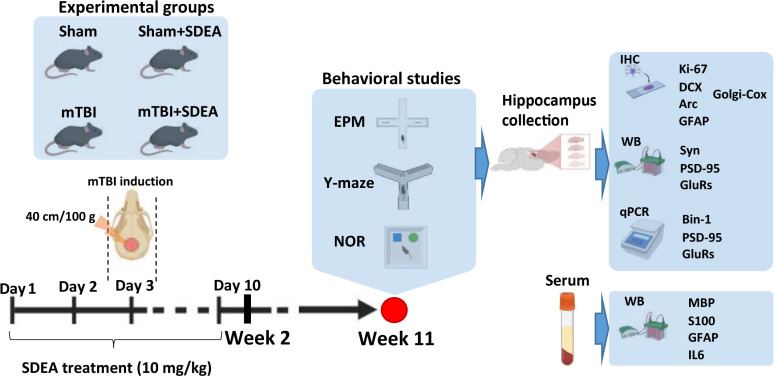
Experimental design and workflow of the study. Mice were divided into four experimental groups: “Sham”, “Sham + SDEA”, “mTBI", and “mTBI + SDEA”. Mild traumatic brain injury (mTBI) was induced using a weight-drop model (40 cm/100 g). SDEA treatment (10 mg/kg) was administered as indicated in the timeline. Behavioral assessments, including the elevated plus maze (EPM), Y-maze, and novel object recognition (NOR) tests, were performed at Week 11. At the end of the experiment (Week 11), animals were euthanized, and the hippocampus was dissected for subsequent molecular, immunohistochemical, and morphological analyses (Golgi-Cox staining).

The test compound (SDEA) was prepared freshly before injections. The emulsion was generated by dispersing the compound in water with continuous vortexing at high speed (3000 rpm for 5 min). Ethanol (1.5% of total injection volume) served as an emulsion stabilizer. An identical ethanol concentration was included in the vehicle solution for control groups. The compound was administered subcutaneously at 10 mg/kg, following a dosing regimen consisting of two pre-treatment days, administration on the day of the injury, and daily injections for the subsequent seven days. Vehicle-treated mice received matched injections of the carrier solution. Four experimental groups were formed: “Sham”—mice underwent all preparatory steps, including anesthesia and positioning under the impact apparatus, but the impact was not delivered. These animals received the vehicle solution containing 1.5% ethanol, serving as baseline controls for both the surgical manipulation and solvent administration (*n* = 17); “Sham + SDEA”—animals received the same sham procedure as described above but were treated with SDEA at 10 mg/kg. This group controlled for the pharmacological effects of the compound in the absence of traumatic injury (*n* = 17); “mTBI”—mice in this group were subjected to a single mild closed-head impact, modeling mTBI, and subsequently received the vehicle solution (*n* = 17); “mTBI + SDEA”—these animals experienced mTBI and were treated with SDEA at 10 mg/kg according to the full dosing schedule (two pre-injury injections, one injection on the day of injury, and daily injections for seven days after) (*n* = 17). This group was used to determine the therapeutic efficacy of SDEA in mitigating mTBI-induced deficits. Behavioral testing was performed in a dedicated cohort (*n* = 14 per group). For gene expression analysis (qPCR), an independent cohort was used to avoid potential confounding effects of behavioral testing on transcriptional readouts. All procedures (mTBI induction, treatment regimen, timing of tissue collection) were identical to the behavioral cohort. The experimental design is schematically summarized in Figure 8.

### 4.5. Behavioral Testing

All behavioral assessments were performed between 10:00 and 16:00 under dim ambient lighting (25–30 lux). Mice were habituated to the testing room for at least 30 min prior to each procedure. All apparatuses were cleaned with 70% ethanol between trials to eliminate olfactory cues.

#### 4.5.1. Y-Maze Spontaneous Alternation Test

Working memory performance was assessed using the spontaneous alternation paradigm in a Y-shaped acrylic maze (30 × 10 × 20 cm), consisting of three identical arms arranged at 120°. All three arms were identical in appearance and odor, and no external cues or bedding differences were introduced, as spontaneous alternation relies on the animal’s intrinsic exploratory behavior rather than explicit arm discrimination. Each mouse was placed at the junction of the three arms and allowed to explore freely for 5 min. An arm entry was counted when the animal completely crossed into an arm with all four paws. The sequence of arm visits was recorded to evaluate spontaneous alternation, defined as consecutive entries into three distinct arms. The test was administered four times at weeks 10–11.

The spontaneous alternation rate (SAR) was calculated as:

*SAR = N/A × 100%* (1)

where *SAR*—the spontaneous alternation rate, *N*—the quantity of consecutive entries into the three nonrepeating arms, *A*—total number of possible alternations (total arm entries—2).

This parameter was used as the primary measure of working memory performance in the Y-maze test.

#### 4.5.2. Novel Object Recognition (NOR) Test

Recognition memory was examined using a standard NOR protocol adapted from Bevins and Besheer [83]. Animals underwent habituation to the testing arena one day prior to the experiment. Training phase: each mouse was placed in the center of the arena containing two identical objects and allowed 10 min of unrestricted exploration. Object interaction was defined as active investigation, including sniffing or touching the object at a distance of <1–2 cm. Testing phase: after a 24 h retention interval, one of the familiar objects was replaced with a novel one. Mice were again placed in the arena for 5 min, and exploration was video-recorded with an overhead camera. To quantify recognition memory, the recognition index (*RI*) and discrimination index (*DI*) were calculated as:

*RI = T_N_/(T_N_ + T_F_)* (2)

*DI = (T_N_ − T_F_)/(T_N_ + T_F_)* (3)

where *T_N_*—time for novel object (s), *T_F_*—time for familiar object (s). Objects and the arena were thoroughly cleaned with 70% ethanol between trials.

These indices were used for subsequent statistical comparisons between experimental groups in the NOR test.

#### 4.5.3. Elevated Plus Maze (EPM)

Anxiety-related behavior was evaluated using a standard elevated plus maze (Panlab, Harvard Apparatus, Holliston, MA, USA) consisting of two open arms (30 × 5 cm, 1 cm ledge) and two closed arms (30 × 5 cm, 15 cm walls), elevated 50 cm above the floor. Each mouse was placed on the central platform facing an open arm and allowed to explore freely for 5 min. Behavior was recorded using a SMART 3.0 video-tracking system (Panlab, Harvard Apparatus, Holliston, MA, USA). The following parameters were quantified: time spent in open arms, time spent in closed arms, and time spent in the center. Increased exploration of open arms was interpreted as reduced anxiety-like behavior, whereas avoidance of open arms indicated heightened anxiety [21].

#### 4.5.4. Tissue Collection

The animals were euthanized under deep anesthesia 11 weeks after surgery, immediately after behavioral testing. Mice were first placed in an induction chamber and anesthetized with 4.5% isoflurane (Laboratories Karizoo, SA, Barcelona, Spain) delivered in 100% oxygen using a rodent anesthetic vaporizer (VetFloTM, Kent Scientific Corporation, Torrington, CT, USA). Brains designated for immunohistochemistry were perfused with PBS followed by 4% paraformaldehyde and postfixed in 4% paraformaldehyde for 24 h. Brains for biochemical analysis were rapidly dissected; hippocampi were isolated on ice, snap-frozen in liquid nitrogen, and stored at −80 °C. Blood was collected via cardiac puncture, centrifuged (3000× *g*, 10 min), and serum was stored at −80 °C.

#### 4.5.5. Immunohistochemistry

After fixation, brains were rinsed in PBS, processed through a standard dehydration and clearing series, and embedded in paraffin blocks. Serial coronal sections (7 µm) were cut using a Leica RM2245 rotary microtome (Leica, Wetzlar, Germany). For immunohistochemical analysis, brain sections were first deparaffinized through a graded xylene and ethanol series and then incubated in 3% hydrogen peroxide for 10 min to suppress endogenous peroxidase activity. After rinsing in 0.1 M phosphate buffer (pH 7.2), sections were transferred into a blocking solution containing 2% bovine serum albumin (Sigma-Aldrich, Darmstadt, Germany) and 0.01% Triton X-100 for 1 h at room temperature to reduce nonspecific antibody binding. Following the blocking step, sections were incubated with primary antibodies for 24 h at 4 °C. The following Abcam primary antibodies were used depending on the target: GFAP (astrocytes): ab7260, 1:1000; Arc (activity-dependent immediate early gene): ab183183, 1:1000; Ki-67 (proliferating cells): ab16667, 1:500; DCX (immature neurons): ab18723, 1:500. Negative controls were processed identically but without addition of primary antibodies. After overnight incubation, sections were washed and incubated with the appropriate HRP-conjugated secondary antibody (Abcam, Cambridge, UK) for 15 min, followed by incubation with streptavidin-HRP (ab64269, Abcam) for 10 min. Visualization was performed using DAB chromogen (Abcam), producing a brown reaction product. Finally, the tissue was rinsed in distilled water, dehydrated through ascending ethanol series, cleared in xylene, and mounted using Dako mounting medium (CS705, Denver, CO, USA).

#### 4.5.6. Image Acquisition and Quantification

Microscopic evaluation was carried out on a Zeiss Axio Imager system equipped with an AxioCam 503 color camera and controlled through ZEN Blue (Carl Zeiss, Oberkochen, Germany) v.3.4.. Digital images were further processed and quantified in ImageJ 1.53t (NIH, Bethesda, MD, USA). Prior to analysis, each micrograph was converted to 8-bit format, and uneven background illumination was corrected using the rolling-ball subtraction algorithm (radius = 50). A region of interest (ROI) corresponding to the CA1 or dentate gyrus was manually outlined based on anatomical landmarks. GFAP immunoreactivity was segmented using a fixed intensity threshold, which was applied uniformly to all images. The percentage of GFAP-positive area was calculated as the fraction of thresholded pixels relative to the total ROI area. The sections were imaged using identical microscope settings (objective, exposure time, and illumination) for all experimental groups. Quantitative analysis was performed using ImageJ software by measuring the GFAP-positive area within CA1 and DG hippocampal regions. All image acquisition and quantification procedures were conducted by an experimenter blinded to group assignment. For quantitative assessment of immunolabeling, every sixth section throughout the hippocampus was selected. All image analyses were performed by an investigator blinded to group assignments to prevent bias.

#### 4.5.7. Golgi-Cox Staining and Dendritic Spine Analysis

Structural plasticity was assessed using the FD Rapid GolgiStain Kit (FD NeuroTechnologies, Columbia, MD, USA). Brains were impregnated for 14 days, cryosectioned at 100 µm, and mounted on gelatin-coated slides. Fully impregnated CA1 pyramidal neurons (apical and basal dendrites) and DG granule neurons were selected based on standard criteria. Dendritic segments (20–40 µm) were imaged at 100× oil immersion, and spine density and morphology (thin, stubby, mushroom, filopodia) were quantified in ImageJ.

#### 4.5.8. Western Blotting

Western blotting was performed to quantify serum levels of GFAP, S100B, MBP, and IL-6, as well as hippocampal expression of Synaptophysin, PSD-95, GluR1, and GluR2. For each experimental group, hippocampal tissue or serum was collected from six biologically independent animals (*n* = 6 per group). Each biological sample was processed individually without pooling. For every sample, two technical replicates (duplicate lanes) were run on the same gel to ensure reliability of signal detection. Serum proteins were precipitated and resuspended in lysis buffer, whereas hippocampal tissue was homogenized manually using a micropestle in ice-cold RIPA buffer supplemented with protease inhibitors (cOmplete™, Sigma-Aldrich, St. Louis, MI, USA). Lysates were centrifuged at 12,000× *g* for 15 min at 4 °C, and the supernatants were collected. Protein concentration was determined with a standard colorimetric assay, after which all samples were normalized to 2 mg/mL. Each lysate was mixed 1:1 with 1× Laemmli sample buffer (Biorad, Hercules, CA, USA) containing 5% β-mercaptoethanol, heated at 94 °C for 5 min, and briefly centrifuged before loading. Equal amounts of protein (60 µg per lane) were separated on Any kDa Mini-PROTEAN precast gels (Biorad, Hercules, CA, USA) alongside a molecular weight ladder (Thermo Fisher Scientific, Waltham, MA, USA). Gels were run at a constant current of 15 mA per gel. Proteins were transferred to PVDF membranes using the Trans-Blot Turbo transfer system (Biorad, Hercules, CA, USA) and the RTA transfer kit under manufacturer-recommended settings. Membranes were incubated in 2% BSA in PBS for 1 h at room temperature and then washed in PBS containing 0.1% Tween-20 (PBS-T). Primary antibodies were applied overnight at 4 °C: GFAP (1:1000, ab7260), S100B (1:1000, ab52642), MBP (1:1000, ab7349), IL-6 (1:1000, ab290735), Synaptophysin (1:500, ab8049), PSD-95 (1:500, ab2723), GluR1 (1:1000, ab31232), GluR2 (1:1000, ab133477), α-tubulin (ab7291, 1:5000, loading control) all from Abcam (Cambridge, UK). After washing (3 × 5 min in PBS-T), membranes were incubated with secondary HRP-conjugated antibodies Anti-Rabbit (Abcam) and Anti-Mouse (Abcam, Cambridge, UK). for 1 h at room temperature, followed by three additional washes. Chemiluminescent substrate (Biorad, Hercules, CA, USA) was applied for 5 min, and signal was visualized with the ChemiDoc imaging system (Biorad, Hercules, CA, USA). Band intensities were quantified in ImageJ (NIH, USA) and normalized to the corresponding loading control. For GluR2 quantification, densitometric analysis was performed only for the band corresponding to the expected molecular weight of GluR2, as indicated by the antibody datasheet. Additional bands were excluded from analysis.

#### 4.5.9. Real-Time PCR

RT-PCR was used to measure hippocampal transcriptional levels of BIN1, GluRs, and PSD95. Because tissue from the primary experimental cohort was fully allocated to histological and protein analyses, qPCR was performed using an additional independent cohort of mice (three animals per group). Hippocampal samples were homogenized using metal beads in a TissueLyser LT system (Qiagen, Hilden, Germany). Total RNA was isolated with the ExtractRNA kit (Evrogen, Moscow, Russia) and further purified using CleanRNA Standard columns (Evrogen, Moscow, Russia). RNA purity and concentration were assessed spectrophotometrically (Thermo Fisher, Waltham, MA, USA). Reverse transcription was performed using the RNAscribe RT kit (Biolambix, Novosibirsk, Russia) according to the manufacturer’s protocol. Real-time PCR was conducted on a CFX96 system (Bio-Rad Laboratories, Hercules, CA, USA) using the SYBR Green I qPCR mix (Evrogen, Moscow, Russia). Reactions (20 μL total volume) contained: 1 μL of cDNA, 0.5 μM forward and reverse primers, SYBR Green master mix. Thermal profile: 95 °C for 10 min, 40 cycles of: 95 °C for 15 s, 67 °C for 40 s, 72 °C for 10 s, melt-curve analysis to confirm single-product amplification. Primers for BIN1, GluRs, and PSD95 were designed in Primer Premier 5 and synthesized by Evrogen (Moscow, Russia). GAPDH and β-actin served as internal reference genes. Relative expression was calculated using the ΔΔCt method, with normalization to the geometric mean of both housekeeping genes.

#### 4.5.10. Statistical Analysis

Data distribution was assessed with Shapiro–Wilk test. Behavioral, biochemical, and histological data were analyzed using two-way ANOVA (factors: Trauma × Treatment), followed by Tukey’s post hoc test. Spine-type-specific datasets were analyzed separately for each dendritic compartment (CA1 apical, CA1 basal, DG) using the same statistical model. Values are presented as mean ± SEM. Statistical significance was set at *p* < 0.05. Graphs were prepared in GraphPad Prism 10 (San Diego, CA, USA).

## 5. Conclusions

N-stearidonylethanolamine effectively counteracts key pathological consequences of mTBI. Treatment attenuated circulating injury biomarkers and astrocytic reactivity, restored PSD-95, AMPA receptor subunits, Arc expression, and rescued CA1 dendritic spine loss while preserving DG structure. These molecular and structural improvements were accompanied by normalization of anxiety-like behavior, whereas recognition and working memory remained intact following mTBI. Together, the findings indicate that SDEA acts as a multi-target modulator of glia-synapse interactions, selectively stabilizing vulnerable CA1 circuits and promoting hippocampal resilience after mild brain trauma.

Limitations: A limitation of the present study is the exclusive use of male animals. Sex-dependent differences in neuroinflammatory responses and synaptic plasticity after traumatic brain injury have been reported, and future studies will be required to assess whether the effects of SDEA extend to female animals.

Although the SGZ of the dentate gyrus represents a neurogenic niche in the adult brain, the present study assessed proliferative activity using Ki-67 without cell-type-specific markers. Therefore, the observed changes should be interpreted as modulation of proliferation within a neurogenesis-associated region rather than direct evidence of altered adult neurogenesis.

Although N-acylethanolamides are highly lipophilic and have been reported to access the central nervous system, direct measurements of SDEA brain penetration were not performed in this study. Therefore, the present findings support functional CNS effects of SDEA but do not directly demonstrate its concentration or structural integrity within brain tissue.

## Data Availability

The raw data supporting the conclusions of this article will be made available by the authors on request.

## Supplementary Materials

The following supporting information can be downloaded at: https://www.mdpi.com/article/10.3390/ijms27010471/s1.

## Abbreviations

The following abbreviations are used in this manuscript:

mTBI	mild traumatic brain injury
CNS	central nervous system
DG	dentate gyrus
CA1	cornu ammonis area 1
PUFA	polyunsaturated fatty acid
NAE	N-acylethanolamide
SDEA	stearidonic acid ethanolamide
DHA	docosahexaenoic acid
EPA	eicosapentaenoic acid
DHEA (synaptamide)	N-docosahexaenoylethanolamide
EPEA	N-eicosapentaenoylethanolamide
PEA	palmitoylethanolamide
GFAP	glial fibrillary acidic protein
S100B	S100 calcium-binding protein B
MBP	myelin basic protein
IL-6	interleukin-6
IL-1β	interleukin-1 beta
TNF-α	tumor necrosis factor alpha
PSD-95	postsynaptic density protein 95
AMPAR	AMPA-type glutamate receptor
GluR1/GluR2	AMPA receptor subunits
NMDAR1/NMDAR2	NMDA receptor subunits
Arc	activity-regulated cytoskeleton-associated protein
BIN1	bridging integrator 1
EPM	elevated plus maze
NOR	novel object recognition
DI	discrimination index
RI	recognition index
SAR	spontaneous alternation rate (Y-maze)
PVDF	polyvinylidene difluoride
ECL	enhanced chemiluminescence
RT-PCR	real-time polymerase chain reaction
SEM	standard error of the mean
ANOVA	analysis of variance
