# Supplementary figures and images for: N-Stearidonoylethanolamine Restores CA1 Synaptic Integrity and Reduces Astrocytic Reactivity After Mild Traumatic Brain Injury
Source: Int J Mol Sci. 2026 Jan 2;27(1):471. doi: 10.3390/ijms27010471 (PMC12786632; doi:10.3390/ijms27010471)

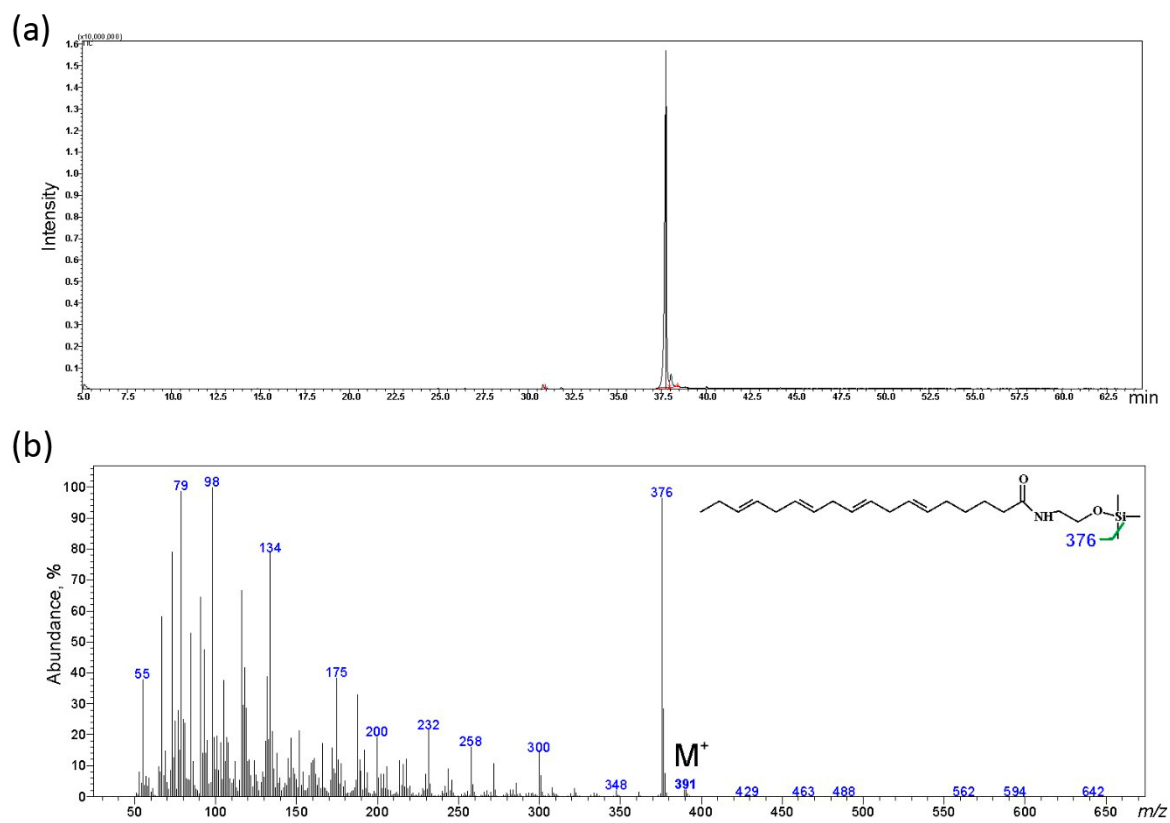

**Figure S1.** Total ion current chromatogram (A) and EI-MS profile (B) of the isolated SDEA compound.

Supplement: Supplementary file 1 [file ijms-27-00471-s001.zip › ijms-4045583 supplementary figureS1.pdf]
